# Enhancement of periodontal tissue regeneration by conditioned media from gingiva-derived or periodontal ligament-derived mesenchymal stem cells: a comparative study in rats

**DOI:** 10.1186/s13287-019-1546-9

**Published:** 2020-02-03

**Authors:** Jiling Qiu, Xiaotong Wang, Haowen Zhou, Chunshu Zhang, Yijia Wang, Jiahui Huang, Meng Liu, Pishan Yang, Aimei Song

**Affiliations:** 1 Department of Periodontology, School and Hospital of Stomatology, Shandong University, No.44-1 Wenhua Road West, Jinan, 250012 Shandong China; 2 Shandong Provincial Key Laboratory of Oral Tissue Regeneration, No.44-1 Wenhua Road West, Jinan, 250012 Shandong China; 3 Shandong Engineering Laboratory for Dental Materials and Oral Tissue Regeneration, No.44-1 Wenhua Road West, Jinan, 250012 Shandong China

**Keywords:** Gingiva-derived mesenchymal stem cells, Periodontal ligament stem cells, Conditioned medium, Periodontal regeneration, Rats

## Abstract

**Background:**

Evidence has demonstrated conditioned medium (CM) from periodontal ligament stem cells (PDLSCs) improved periodontal regeneration. Gingival mesenchymal stem cells (GMSCs) have been considered an alternative strategy for regenerative medicine. To determine whether GMSC-CM could promote periodontal wound healing, we compared the effects of GMSC-CM and PDLSC-CM on periodontal regeneration and the underlying mechanisms in rat periodontal defects.

**Methods:**

Cell-free CMs were collected from PDLSCs, GMSCs, and gingival fibroblasts (GFs) using ultracentrifugation (100-fold concentration). Periodontal defects were created on the buccal side of the first molar in the left mandible of 90 rats by a surgical method. Collagen membranes loaded with concentrated CMs (α-MEM, GF-CM, GMSC-CM, PDLSC-CM) were transplanted into periodontal defects. After 1, 2, and 4 weeks, the animals were sacrificed and specimens including the first molar and the surrounding tissues were separated and decalcified. Hematoxylin-eosin and Masson’s trichrome staining were performed to evaluate periodontal regeneration. Immunohistochemical staining for tumor necrosis factor (TNF)-α, interleukin (IL)-1β, and IL-10 was conducted to analyze inflammation. Immunohistochemistry of BSP-II and Runx2 was performed to analyze osteoblast differentiation.

**Results:**

Histological analysis showed the amount of newly formed periodontal tissue was significantly higher in both the GMSC-CM and PDLSC-CM groups than in the other groups, with no significant difference between these two groups. At 1 and 2 weeks, the expression levels of TNF-α and IL-1β were significantly lower in the GMSC-CM and PDLSC-CM groups than in the other three groups, while there was no significant difference between these two groups. IL-10 expression was significantly higher in the GMSC-CM group than in the PDLSC-CM group and the other three groups. At 1, 2, and 4 weeks, BSP-II and Runx2 expressions were significantly higher in the GMSC-CM and PDLSC-CM groups than in the other three groups, with no significant difference between the two groups.

**Conclusions:**

Our results demonstrate that GMSC-CM transplantation can significantly promote periodontal regeneration in rats and achieve the same effect as PDLSC-CM. The mechanism of periodontal regeneration may involve the regulation of inflammatory factors and the promotion of osteogenic differentiation of bone progenitor cells in the wound region by CMs from MSCs.

## Background

Periodontitis is a chronic inflammatory disease that involves the destruction of connective tissue attachments and alveolar bone resorption [1]. Progressive destruction of alveolar bone supporting teeth can lead to tooth loosening and displacement, gingival recession, and eventually tooth loss [2]. In China, the incidence of periodontitis among adults over 35 years old is up to 40%. The ultimate goal of treatments for periodontitis is the regeneration of damaged periodontal tissue [3, 4].

Mesenchymal stem cells (MSCs) are nonhematopoietic stromal cells that can be isolated from various adult tissues, such as the bone marrow, placental, adipose, umbilical cord, periodontal ligament, and gingival tissue [5, 6]. The capacity of MSCs to differentiate into multipotent cells [7, 8] has advanced them to the forefront in regenerative medicine [9, 10]. An increasing number of reports have indicated that, apart from their multi-differentiation potential, the paracrine pathway might be the principal mechanism by which MSCs contribute to tissue regeneration [11, 12].

Conditioned medium generated by MSC culture contains growth factors, cytokines, and other active substances [13]. MSC-CM transplantation, as a cell-free technique, is more convenient and safer to apply and has much greater potential for clinical translation than MSC transplantation [14, 15]. A variety of studies have indicated that MSC-CM has immunomodulating, angiogenesis-promoting, and cell growth-supporting properties, and MSC-CM has been reported to enhance regeneration in many animal wound models [16], including periodontal defect models [17, 18].

Periodontal ligament stem cells (PDLSCs), as the adult stem cell population in tooth-supporting tissues [19], are the most widely studied and used for periodontal tissue regeneration [20, 21]. Transplantation of PDLSC-CM has been documented to result in considerable new connective attachment and alveolar bone formation. However, to obtain PDLSCs in vitro, multiple periodontal membranes of extracted teeth are required, and the success rate of culture is very low [22]. A long amount of time (1 to 2 months) is required to obtain the large number of PDLSCs necessary for in vivo transplantation [23, 24]. Therefore, the widespread application of PDLSCs in periodontal therapy is severely influenced due to its limited access.

Therefore, we sought to explore CMs from other stem cells for use in regenerating lost periodontal tissue. Gingiva-derived mesenchymal stem cells (GMSCs) can be isolated from gingival connective tissue and have been confirmed to have a self-renewal capacity, multi-differentiation potential, and strong immunomodulatory properties [25]. GMSCs are more readily available than PDLSCs and other MSCs due to their abundant sources without requiring tooth extraction [26, 27]. The gingiva collection procedure is minimally invasive and enables scarless wound healing [28]. Moreover, some studies have shown that GMSCs have superior anti-inflammatory and immunomodulatory functions [29, 30]. There have been reports indicating that transplantation of GMSCs contributes to bone tissue regeneration in animal bone defect models [31, 32] and periodontal regeneration [33]. Thus, GMSCs may be promising for use in future regenerative medicine as a readily available stem cell source.

Although there are a few reports involving the transplantation of GMSC-CM for tissue regeneration [34, 35], its use in periodontal regeneration has not been reported. Therefore, in this study, we compared the effect of GMSC-CM on periodontal regeneration and the underlying mechanism with the effect of PDLSC-CM by using a periodontal defect model in rats, aiming to provide a theoretical basis for the application of GMSC-CM in periodontal regeneration in the future.

## Methods

### Tissue sources

Healthy gingival tissue samples were obtained from three donors (18 to 25 years old) who provided informed consent undergoing mandibular third molar extraction at the Hospital of Stomatology, Shandong University. Tissue specimens were collected following the approval of Human Research Projects, the Ethics Committee of School of Stomatology, Shandong University (No. GR201407).

### Cell culture

#### Human GMSC culture and identification

Human GMSCs were isolated from healthy gingival tissues by the finite dilution method according to the report by Du [36]. Briefly, each gingival tissue sample was diced into 1-mm^3^ pieces, and culture medium was added. Approximately 7 to 10 days later, spindle-like cells grew out from the tissues, and the cells were collected with trypsin. Individual cells were plated in 10-cm culture dishes (3300 cells/dish). After 10 to 15 days, single-cell-derived colonies were collected and subcultured. Passage 4 cells were used for GMSC identification and the following experiments. During the experiment, cells were cultured in α-minimal essential medium (α-MEM; Sigma-Aldrich, St. Louis, MO, USA) containing 20% fetal bovine serum (FBS; Biological Industries, Kibbutz Beit-Haemek, Israel), 100 U/ml penicillin, and 0.1 mg/ml streptomycin at 37 °C with 5% CO_2_. The complete medium was replaced every 3 days.

#### Characterization of GMSCs

For MSC-associated surface antigen identification, the above cells were detached into single-cell suspensions (10^6^/ml) in phosphate-buffered saline (PBS) and then incubated with fluorescein isothiocyanate-conjugated mouse monoclonal antibodies (10 μg/ml) specific for human CD35, CD45, CD90, CD105, and CD44 (BioLegend, San Diego, CA, USA) for 1 h on ice away from light. The cells were then washed with PBS, and the suspensions were subjected to flow cytometry (BD Biosciences, Franklin Lakes, NJ, USA).

For multidifferential potential identification, cells were cultured in osteogenic (1 × 10^−8^ mol/l hexadecadrol, 5 mmol/l sodium β-glycerophosphate, 50 mg/l vitamin C, and 10% FBS in α-MEM) or adipogenic (0.5 μmol/l hexadecadrol, 60 μmol/l indomethacin, 0.5 mmol/l isobutyl methylxanthine, 10 mg/l bovine insulin, and 10% FBS in α-MEM) media for 28 and 21 days with the media changed every 3 days. Cells were then washed with PBS three times and fixed in 4% paraformaldehyde. Osteogenic cultures were stained with Alizarin Red (Solarbio, Beijing, China), and adipogenic cultures were stained with Oil Red O (Solarbio).

#### Human gingival fibroblast culture

Instead of the limiting dilution method for GMSCs, cells growing out from the cultured gingival tissue were collected with trypsin and subcultured six times. Gingival fibroblasts (GFs) were obtained at passage 7.

#### PDLSC culture

PDLSCs for isolation and culture were kindly provided by Chunshu Zhang [37].

#### Preparation and concentration of CMs

GMSCs, PDLSCs, and GFs were cultured to 80% confluency in 10% FBS complete medium. Then, the medium was replaced by serum-free α-MEM, and the cells were cultured for another 48 h at 37 °C under 5% CO_2_. The supernatants of the GMSC, PDLSC, and GF groups were collected, centrifuged at 173*g* for 5 min, and then passed through 0.22-μm filters to obtain CMs. Then, the acquired CMs were concentrated 100-fold using ultrafiltration centrifuge tubes (Ultra-15 10 kD centrifugal filter, EMD Millipore, Billerica, MA, USA) at 5000*g* and 4 °C for 40 min according to the manufacturer’s instructions. Control CM was collected from serum-free α-MEM, incubated for 48 h at 37 °C under 5% CO_2_, and concentrated as mentioned above. Then, the bicinchoninic acid (BCA) (Solarbio) method was used to determine the protein concentration in the CMs. All concentrated CMs were subpackaged and stored at − 80 °C.

### Animal experiment

#### Experimental animals

Ninety male Wistar rats (6 to 7 weeks of age, weighing 200 to 230 g) were procured from the Experimental Animal Center, Shandong University. Rats were housed in individual ventilated cages and provided ad libitum access to both food and water. After 1 week, rats were allocated randomly into five groups: a control group, an α-MEM group, a GF-CM group, a GMSC-CM group, and a PDLSC-CM group, for the subsequent study. All animal experiments were approved by the Ethics Committee of School of Dentistry, Shandong University (No. GD201714).

#### Establishment of the rat periodontal defect model

After 1 week of acclimation, rats were anesthetized with an intraperitoneal injection of pentobarbital sodium (40 mg/kg). The periodontal defect model was established according to Nagata et al. [17] with slight modification of the root surface treatment. Briefly, the left mandible buccal plate was exposed through an extraoral incision. Then, the buccal bone, horizontally from the mesial root of the first mandibular molar to the mesial root of the second mandibular molar and vertically from the most coronal aspect of the alveolar crest to the apical root, was carefully removed by turbomachinery to expose the surfaces of the mesial, middle, and distal roots of the first molar. Specifically, all exposed root surfaces were completely debrided with a mini-Gracey curette (Hu-Fridy, USA) to remove the periodontal ligament and the cementum. The defect was approximately 3 mm wide, 2 mm in height, and 1 mm in depth (see Additional file 1).

#### Transplantation of conditioned media

After the defects were thoroughly rinsed with sterilized normal saline, blood was fulfilled. Each defect received one type of CM loaded with resorbable collagen scaffolds (Bio-Gide, Geistlich Biomaterials, Wolhusen, Switzerland). The collagen membrane was cut to a size of 2 mm × 3 mm and immersed in concentrated α-MEM, GF-CM, GMSC-CM, or PDLSC-CM for 12 h at 4 °C before surgery. In the blank control group, the same sized membranes were dipped in sterilize normal saline under the same conditions. After the scaffolds were transplanted into the defect, the buccal masseter and the skin were repositioned to cover the defect and sutured with 5-0 and 3-0 surgical silks, respectively. All animals received soft food and injections of preventive antibiotics for 3 days, followed by normal food and water. The rats were sacrificed after 1, 2, and 4 weeks, and the mandibles were isolated for further study.

### Histology and immunohistochemistry

The animals were sacrificed under anesthesia, and the specimens, including the first molar and its surrounding periodontal tissue, were separated, fixed in 4% paraformaldehyde for 48 h, and decalcified in 12.5% ethylene diamine tetraacetic acid (EDTA, Solarbio) (pH 7.3–7.5) for up to 8 weeks. After dehydration and hyalinization, the specimens were embedded in paraffin. A series of buccal-lingual sections (5 μm thick) paralleling the long axis of the teeth were obtained. Sections that passed through the center of the middle root of the first molar were stained with hematoxylin-eosin (HE) (Solarbio) and modified Masson’s trichrome (Solarbio) and were then subjected to immunohistochemistry according to the manufacturer’s instructions. The antibodies used were as follows: mouse monoclonal anti-bone sialoprotein (BSP)-II (1:100, Santa Cruz Biotechnology, Dallas, TX, USA), mouse monoclonal anti-Runt-related transcription factor 2 (Runx2) (1:200, Abcam, Cambridge, MA, USA), rabbit polyclonal anti-tumor necrosis factor (TNF)-α (1:100), anti-interleukin (IL)-1ß (1:200, Abcam), and anti-IL-10 (1:100, Abcam) primary antibodies. A biotin-labeled goat anti-mouse/rabbit IgG complex was the secondary antibody (SPlink detection kit; ZSGB-BioTech, Beijing, China). Immunohistochemical staining was performed with a diaminobenzidine kit (ZSGB, Bio Tech).

### Histological observation and measurements

Periodontal tissue regeneration was observed with a light microscope (Olympus, Tokyo, Japan). The area of newly formed alveolar bone was calculated by Image-Pro Plus 6.0 software (Media Cybernetics, Rockville, MD, USA). For the immunohistochemistry analysis, images were obtained with a light microscope (Olympus). The integrated optical densities of BSP II, TNF-α, IL-1β, and IL-10 positive stainings were measured by Image-Pro Plus 6.0 software, while Runx2-positive cells were counted by microscope observation.

### Statistical analyses

Statistical analyses were conducted with Prism version 6.01 software (GraphPad, La Jolla, CA, USA). Data were expressed as the mean ± standard deviation. Differences in new alveolar bone area and immunohistochemical density between groups were evaluated by one-way ANOVA, and the mean value of each group was compared using the Student–Newman–Keuls (SNK) test. Statistical significance was accepted with a *P* value less than 0.05.

## Results

### Isolation and identification of GMSCs

**Fig. 1:**
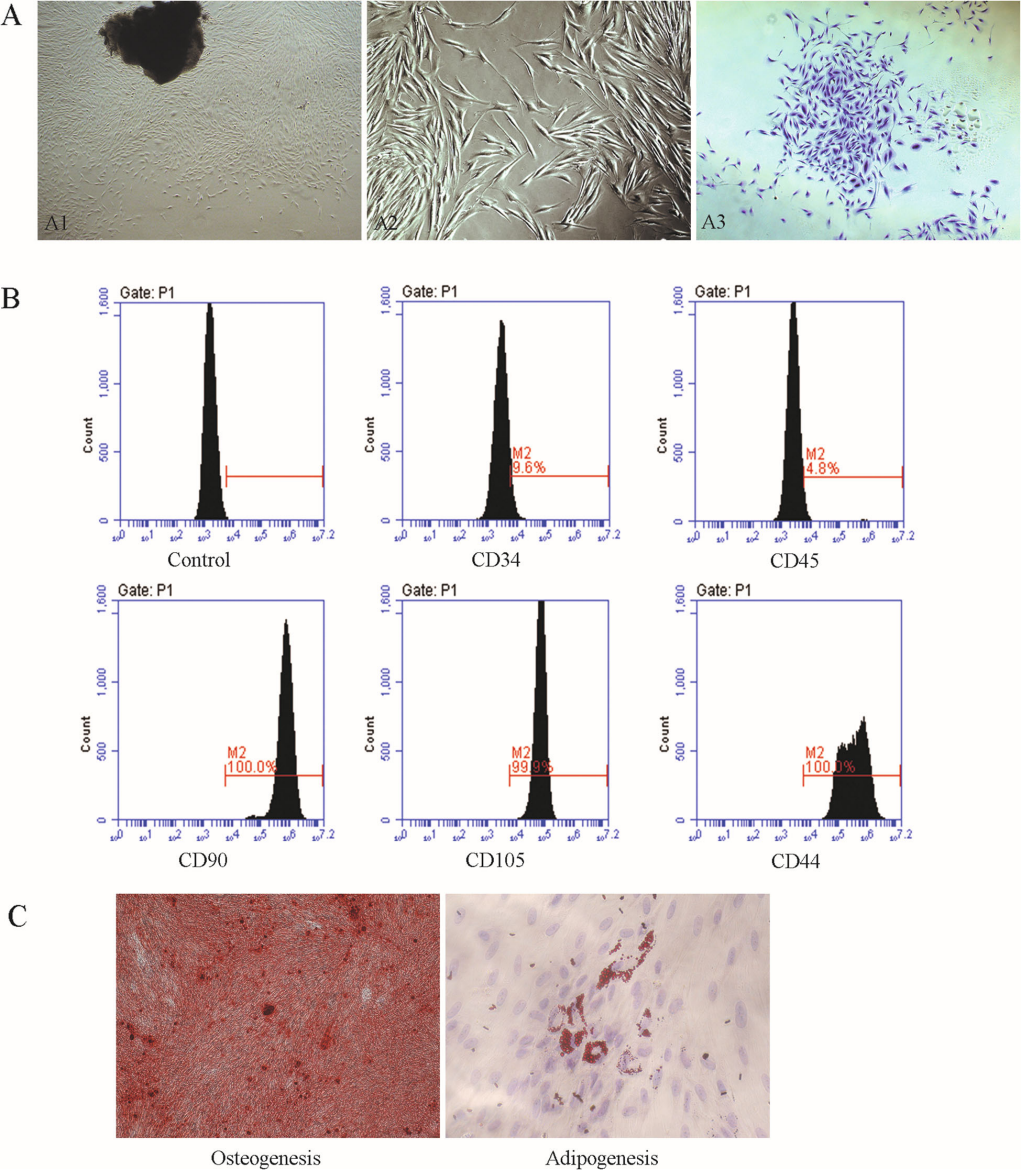
Isolation and characterization of gingiva-derived mesenchymal stem cells (GMSCs). **A** Isolation of GMSCs. A1 Morphology of fibroblast-like cells at passage 0 (40×); A2 Spindle-shaped cells at passage 1 (100×); A3 clonogenic colonies of GMSCs at passage 2 (100×, stained with crystal violet). **B** Expression of representative surface markers determined by flow cytometric analysis. **C** Osteogenic (osteogenesis, 40×) and adipogenic (adipogenesis, 200×) differentiation capacity of GMSCs in vitro

Spindle-shaped cells grew out from the transplanted gingival pieces and reached 80–90% confluency at 10 to 14 days in the culture plates (Fig. 1 A1, A2). Colonies (stained with crystal violet) were formed by single-cell suspension after 15 days, and cells showed a fibroblast-like morphology (Fig. 1 A3). The cells cultured from the fibroblastic colony-forming units were negative for expression of the hematopoietic markers CD34 (9.6%) and CD45 (4.8%) and positive for expression of the MSC-associated surface markers CD90 (100%), CD105 (99.9%), and CD44 (100%) (Fig. 1B). After induction with osteogenic medium for 28 days, the cultured cells exhibited multilayered growth, and calcium deposits stained with Alizarin Red were observed microscopically (Fig. 1C), indicating that the cultured cells showed the potential for osteogenic differentiation. After induction for 21 days with adipogenic medium, the cultured cells could produce microscopic fat droplets as demonstrated by Oil Red O staining (Fig. 1C), which indicated that the cultured cells could be induced to differentiate into adipocytes. Taken together, the above results verified that we successfully isolated GMSCs.

### Periodontal tissue regeneration in periodontal defects

**Fig. 2:**
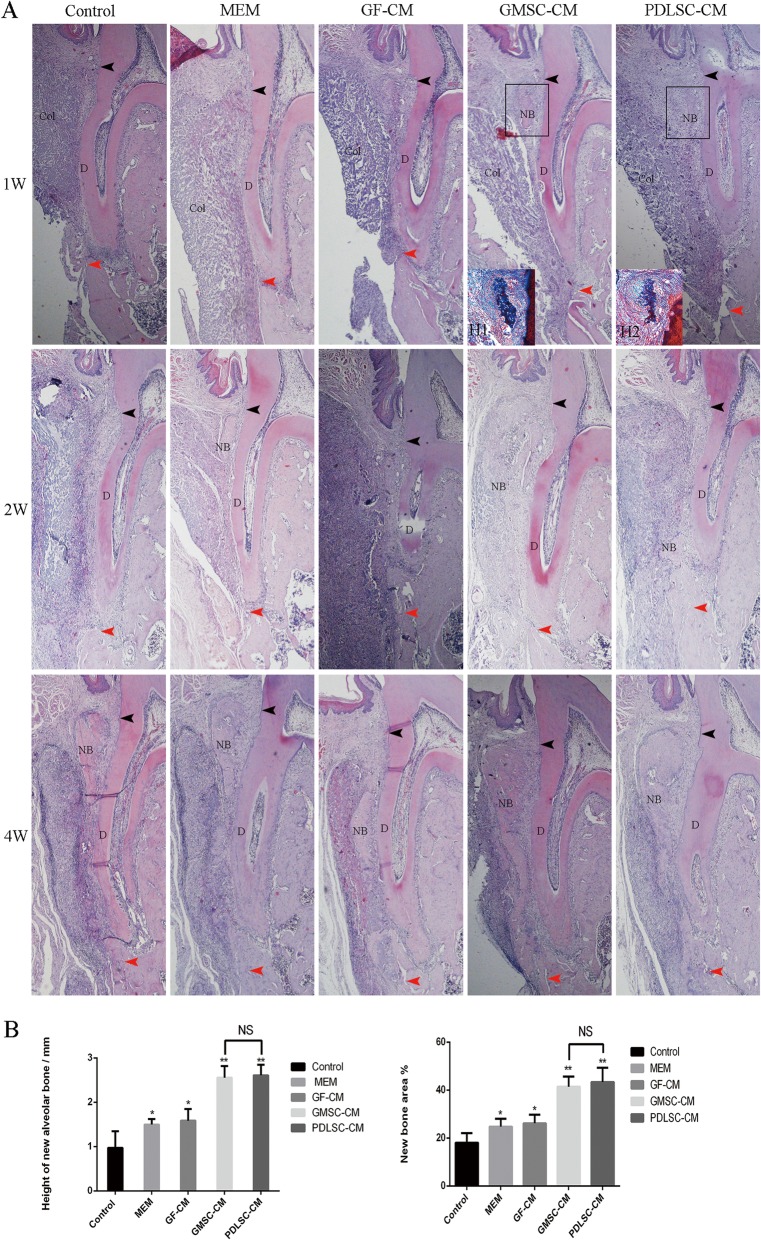
New alveolar bone formation observed by hematoxylin-eosin staining. **A** Representative images (40×) of new alveolar bone at 1, 2, and 4 weeks. NB, new alveolar bone; Col, collagen membrane; D, mandibular first molar tooth; black arrows, coronal limit of the defect; red arrows, apical limit of the defect. H1: Masson staining of GMSC-CM group (200×), H2: Masson staining of PDLSC-CM group (200×). **B** Statistical analysis of the area percentage and height of new alveolar bone in bone defects at 4 weeks. *P < 0.05, **P < 0.01 vs. control groups; NS, no statistical significance

**Fig. 3:**
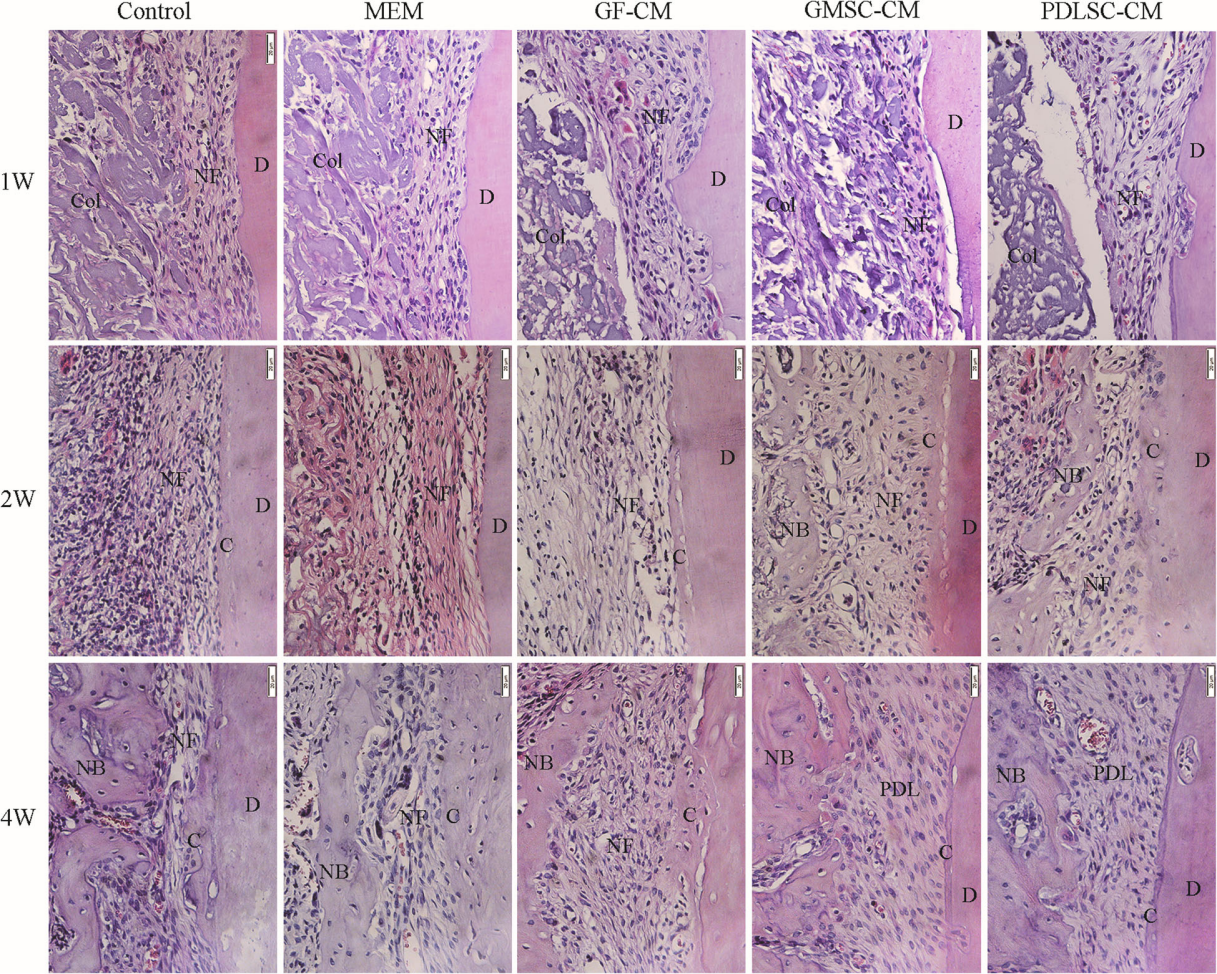
Higher magnification images of new periodontal tissue formation observed by hematoxylin-eosin staining. Representative images (400×) of new periodontal tissue formation at 1, 2, and 4 weeks. NB, new alveolar bone; D, mandibular first molar tooth; NF, new fiber; C, new cementum; PDL, new periodontal ligament

**Fig. 4:**
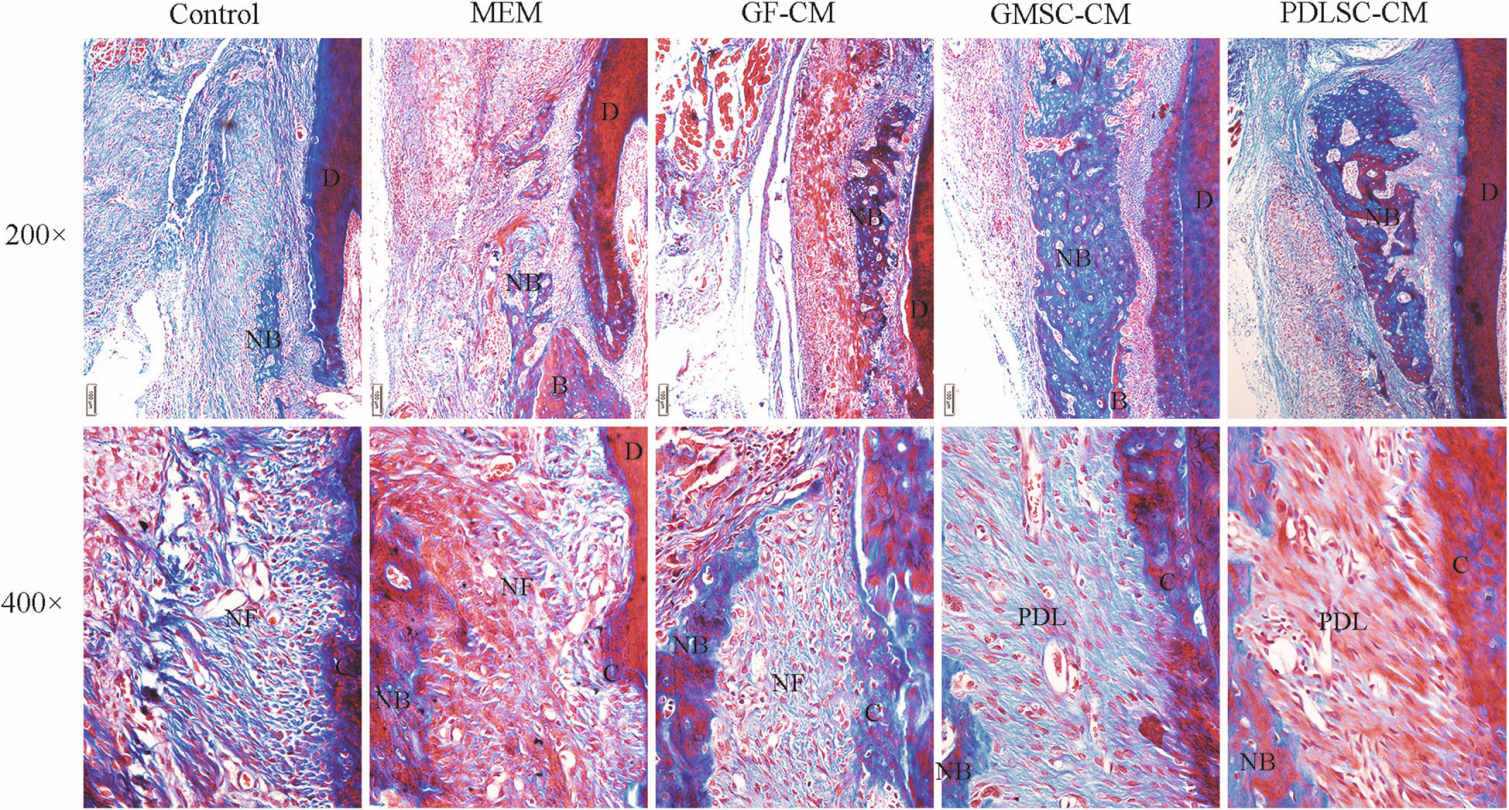
Periodontal regeneration observed by Masson’s trichrome staining. The top panel (200×): new alveolar bone formation observed by Masson’s trichrome staining. Representative images of new alveolar bone at 4 weeks. NB, new alveolar bone (blue and red); B, original bone (red); D, mandibular first molar tooth. The bottom panel (400×): new fibrous tissue formation observed by Masson’s trichrome staining at 4 weeks. NF, new fibrous tissue; PDL, new periodontal ligament; NB, new bone; D, mandibular first molar tooth; C, new cementum

The goal of periodontal therapy is the complete regeneration of periodontal tissue (new alveolar bone, cementum, and inserted periodontal ligament), which remains a great challenge in the treatment of periodontitis. The regeneration of periodontal attachment requires not only alveolar bone formation but also cementum formation in which the newly formed periodontal ligament can be anchored. In this study, periodontal defects were created on the buccal side of the first molar in the left mandible of rats by a surgical method, and physiological saline, α-MEM, GF-CM, GMSC-CM, or PDLSC-CM was transplanted with a resorbable bilayer membrane, as described in the “Methods” section. To evaluate the effects of CMs on periodontal tissue regeneration, a newly formed bone in the periodontal defects was observed and measured in low-magnification HE-stained histological sections (Fig. 2) and in high-magnification Masson-stained sections at 4 weeks (Fig. 4), and periodontal ligament and cementum regeneration were observed under high magnification in each group (Figs. 3 and 4).

At 1 week, the defect area of each group was well filled with collagen membrane. In the apical region, the membrane just reached the old alveolar bone. In the coronal region, the membrane was located beneath the gingival connective tissue (top panel of Fig. 2A). A layer of connective tissue infiltrated with inflammatory cells and fibroblasts was observed between the root surface and the transplanted collagen membrane. These newly formed tissues were not well organized, and there was no much difference among the five groups. The root cementum was removed, and there was no newly formed cementum in any of the five groups at this time (top panel of Figs. 2A and 3). A few of specimens in GMSC-CM and PDLSC-CM groups showed the islet-like new bone in the coronal region of the defect (top panel of Fig. 2A, Table 1; see Additional file 2). The newly formed bone-like tissue was distinguished which was demonstrated by Masson staining (H1, H2, embedded in the top panel of Fig. 2A; see Additional file 3 and Additional file 4).

**Table 1:** The distribution of specimens with new bone formation in each group

Groups	Control	MEM	GF-CM	GMSC-CM	PDLSC-CM
1 week	0	0	0	2	1
2 weeks	2	2	3	6	6

At 2 weeks, the collagen membrane was partially absorbed, and the infiltration of inflammatory cells was reduced. Newly formed bone-like tissue was located in the coronal region of the defect and between the root surface and the collagen membrane (middle panel of Fig. 2A). In the GMSC-CM and PDLSC-CM groups, with the exception of the new bone formed in the coronal region, there was still more newly formed loose trabeculae bone growing coronally from the apical old bone (middle panel of Fig. 2A). New loose trabeculae bone could be observed in each group, but not in all specimens. The new bone formation observed in each group is listed in Table 1. The connective tissue, which separated the new bone from the root surface, became wider and more orderly when compared with 1 week. However, negligible differences in the morphology of the connective tissue were found among the groups (middle panel of Fig. 3). By high magnification, newly formed cellular cementum-like tissue could be observed along the root surfaces in each group, but not in all specimens. There were more new cementum formation specimens in the GMSC-CM and PDLSC-CM groups than in the other three groups. The new cementum formation allocated in each group was listed in Table 2.

**Table 2:** The number of specimens with new cementum formation in each group

Groups	Control	MEM	GF-CM	GMSC-CM	PDLSC-CM
1 week	0	0	0	0	0
2 weeks	1	1	2	4	5
4 weeks	4	5	5	6	6

At 4 weeks, the implanted collagen membrane remained clearly visible, while inflammatory infiltration was further reduced (bottom panel of Fig. 2A). The newly formed bone became apparently wider and denser in the coronal region, while in the apical region, some specimens still showed porous trabecular bone and were mainly stained blue in the Masson-stained sections (top panel of Fig. 4), which indicated that the bone was still in the process of maturity. In the GMSC-CM and PDLSC-CM groups, the height of the new alveolar bone was significantly increased, and the new bone almost fully filled the whole defect. All specimens showed bone formation, and there was more organized connective tissue between the newly formed bone and the root surface. No ankylosis, namely, direct bone-root contact, was found in our experiment. No significant differences in the area or height of neonatal alveolar bone were observed between the GMSC-CM group and the PDLSC-CM group. However, the area percentage and height of neonatal alveolar bone were significantly higher in both the GMSC-CM group and the PDLSC-CM group than those in the other three groups (Fig. 2B). New cementum-like tissue and periodontal fibers could be observed in most of the specimens. In the GMSC-CM and PDLSC-CM groups, periodontal fibers were inserted into the newly formed acellular cementum or cellular cementum, then oblique coronally and inserted into the newly formed alveolar bone (bottom panel of Figs. 3 and 4), which was similar to the periodontal structure in the intact lingual side. In the other three groups, the root surface was mainly covered by cellular cementum, and the connective tissue was less orderly aligned than those in the GMSC-CM and PDLSC-CM groups. Also, there was always a very thin space between the root dentin and the newly formed cementum (bottom panel of Figs. 3 and 4).

### Expression of osteogenesis-related markers in periodontal defects

**Fig. 5:**
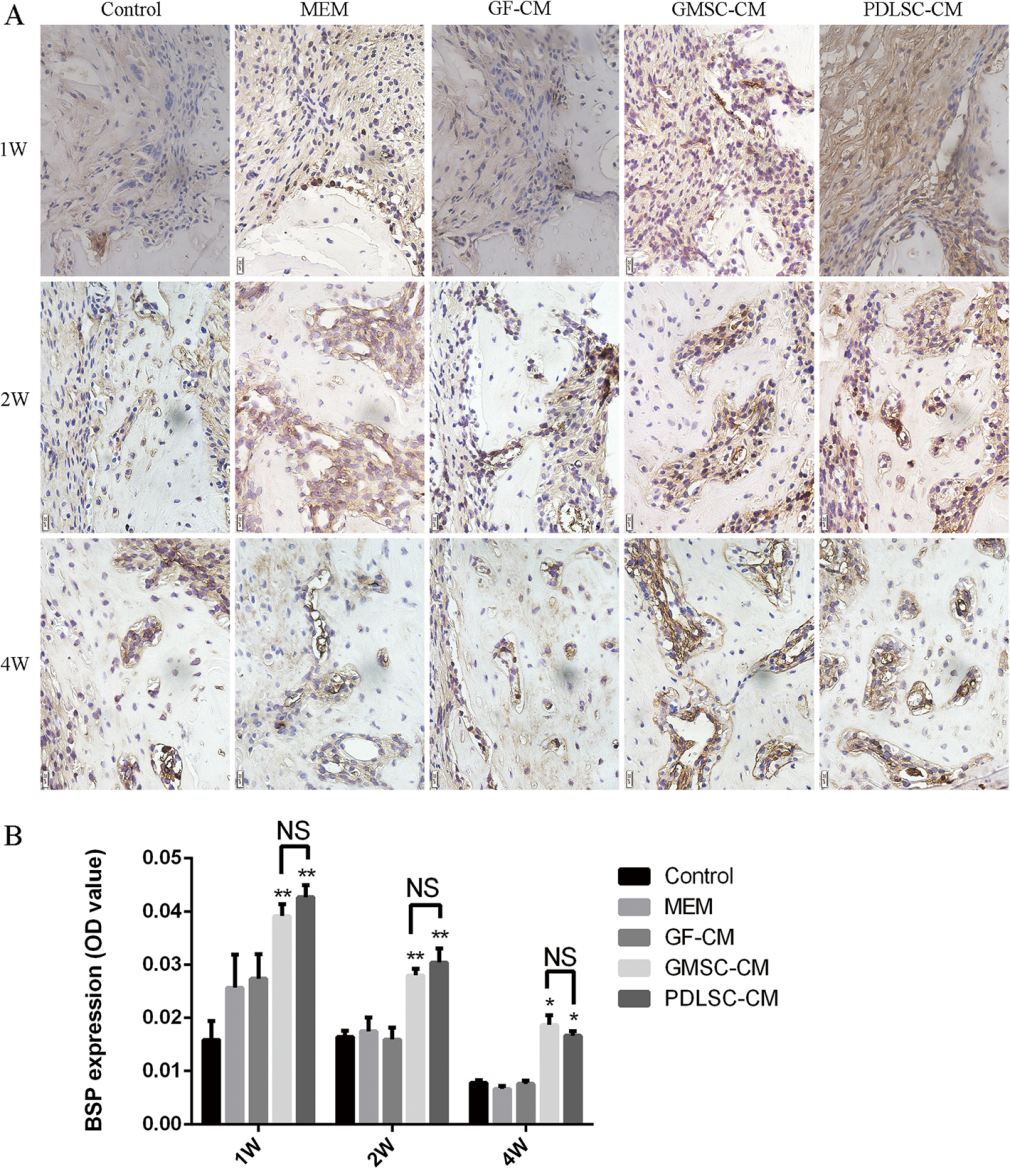
Effect of conditioned media on the osteogenesis-related marker BSPII. **A** Representative images of immunohistochemistry of BSPII (400×) at 1, 2, and 4 weeks. **B** Relative quantification of BSPII immunohistochemical staining. *P < 0.05, **P < 0.01 vs. control groups; NS, no statistical significance

**Fig. 6:**
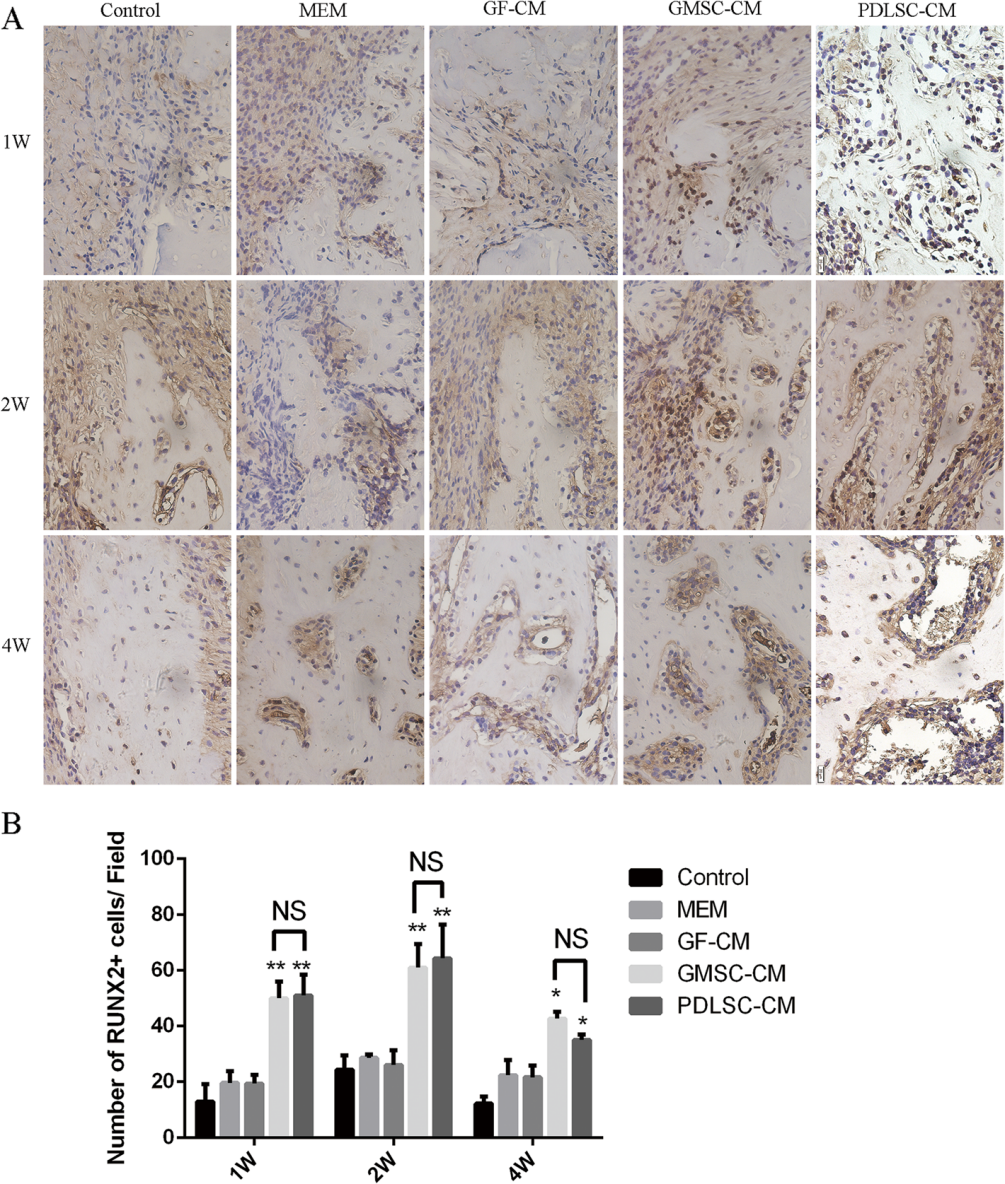
Effect of conditioned media on the osteogenesis-related marker Runx2. **A** Representative images of immunohistochemistry of Runx2 (400×) at 1, 2, and 4 weeks. **B** Relative quantification of Runx2 immunohistochemical staining. *P < 0.05, **P < 0.01 vs. control groups; NS, no statistical significance

Since the volume of regenerated periodontal tissue was dependent on the type of conditioned mediums and as it has been documented that CMs generated by MSC culture contains growth factors, cytokines, and other active substances through which MSC-CM can enhance bone regeneration [17, 18], it is conceivable that the promotion of osteogenesis/cementogenesis might explain the regenerative function of PDLSC-CM and GMSC-CM. Thus, we evaluated the effect of CMs on osteogenesis/cementogenesis by BSPII and Runx2 immunohistochemical staining. The results showed that at 1 week (Fig. 5A top panel), abundant yellow-brown BSPII-positive cells were observed in the marrow between the new bone in the PDLSC-CM and GMSC-CM groups. By 2 and 4 weeks, the BSPII-positive cells had gradually diminished and were mainly observed at the trabecular edge of the new bone (Fig. 5A middle and bottom panel). Runx2-positive cells were also observed around the newly formed alveolar bone. The number of Runx2-positive cells in each group was higher at 2 weeks than at 1 week. However, by 4 weeks, the number of Runx2-positive cells had decreased in each group, and these cells were mainly observed at the edge of the new trabecular bone (Fig. 6A).

At 1, 2, and 4 weeks, the GMSC-CM and PDLSC-CM groups exhibited significantly higher expression of BSPII (Fig. 5B) and Runx2 (Fig. 6B) than the other three groups (*P* < 0.05), while there were no significant differences between the GMSC-CM and PDLSC-CM groups.

### Inflammation modulation after CM transplantation

**Fig. 7:**
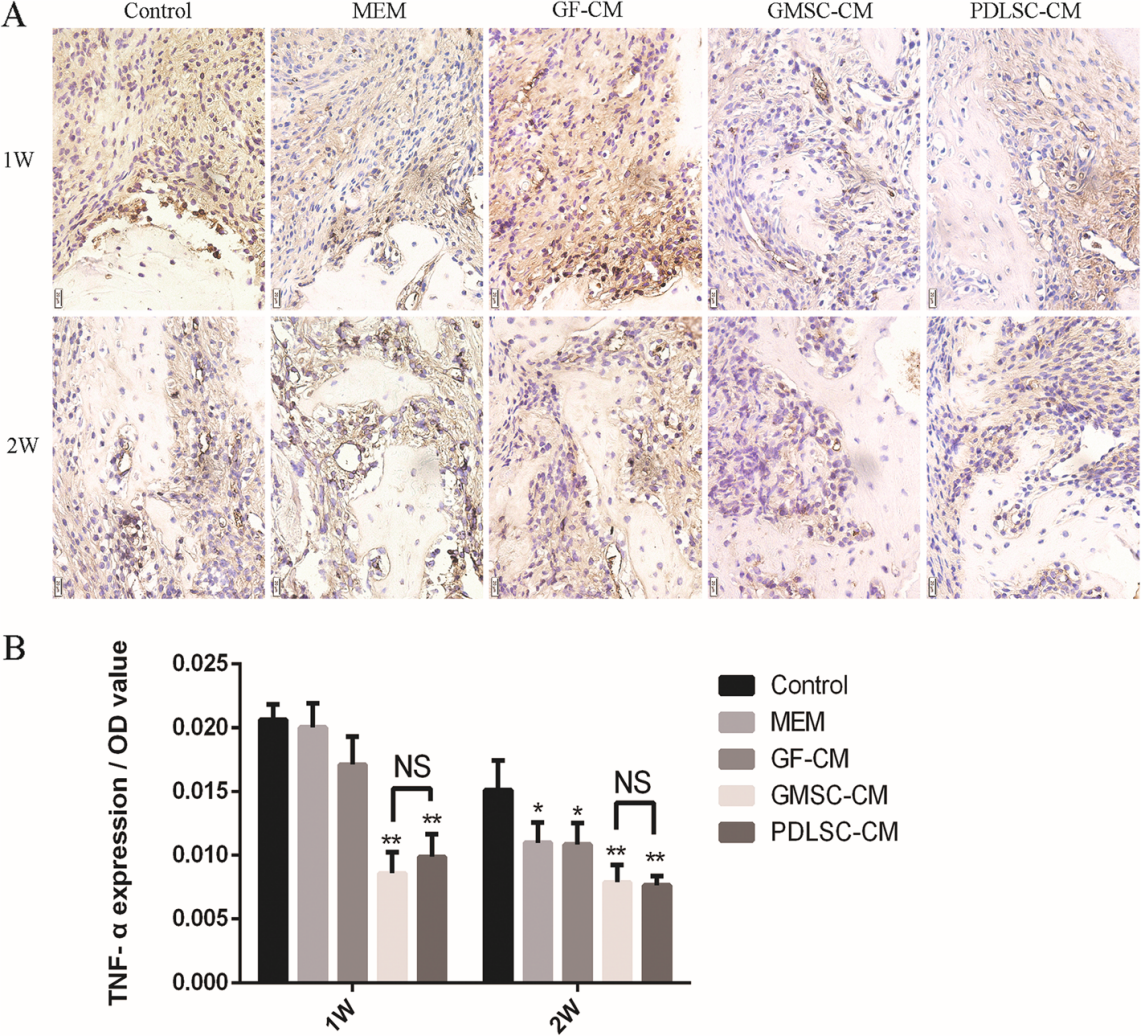
Effect of conditioned media on the osteogenesis-related marker TNF-α. **A** Representative images of immunohistochemistry of TNF-α (400×) at 1 and 2 weeks. **B** Relative quantification of TNF-α immunohistochemical staining. *P < 0.05, **P < 0.01 vs. control groups; NS, no statistical significance

**Fig. 8:**
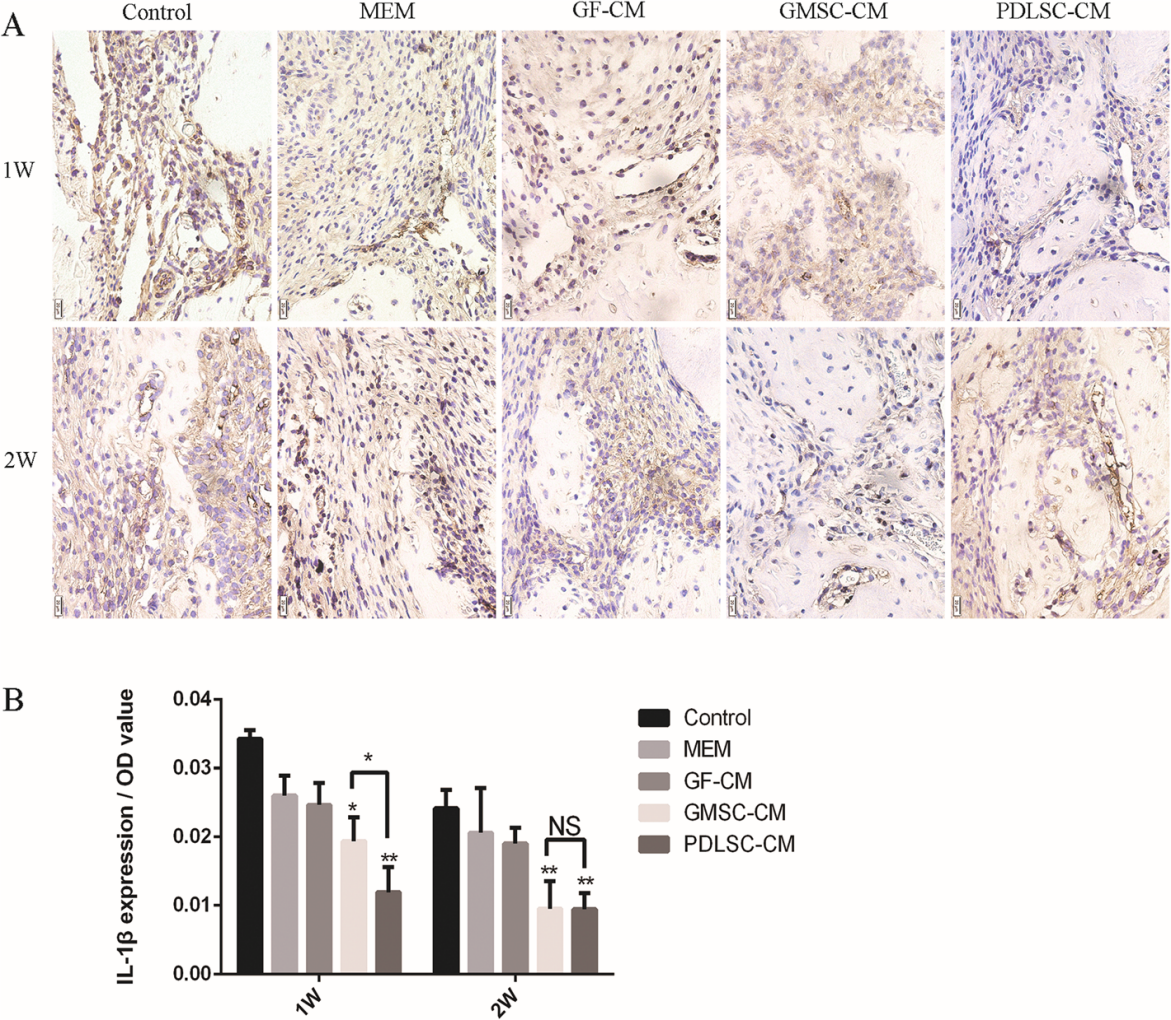
Effect of conditioned media on the osteogenesis-related marker IL-1β. **A** Representative images of immunohistochemistry of IL-1β (400×) at 1 and 2 weeks. **B** Relative quantification of IL-1β immunohistochemical staining. *P < 0.05, **P < 0.01 vs. control group; NS, no statistical significance

**Fig. 9:**
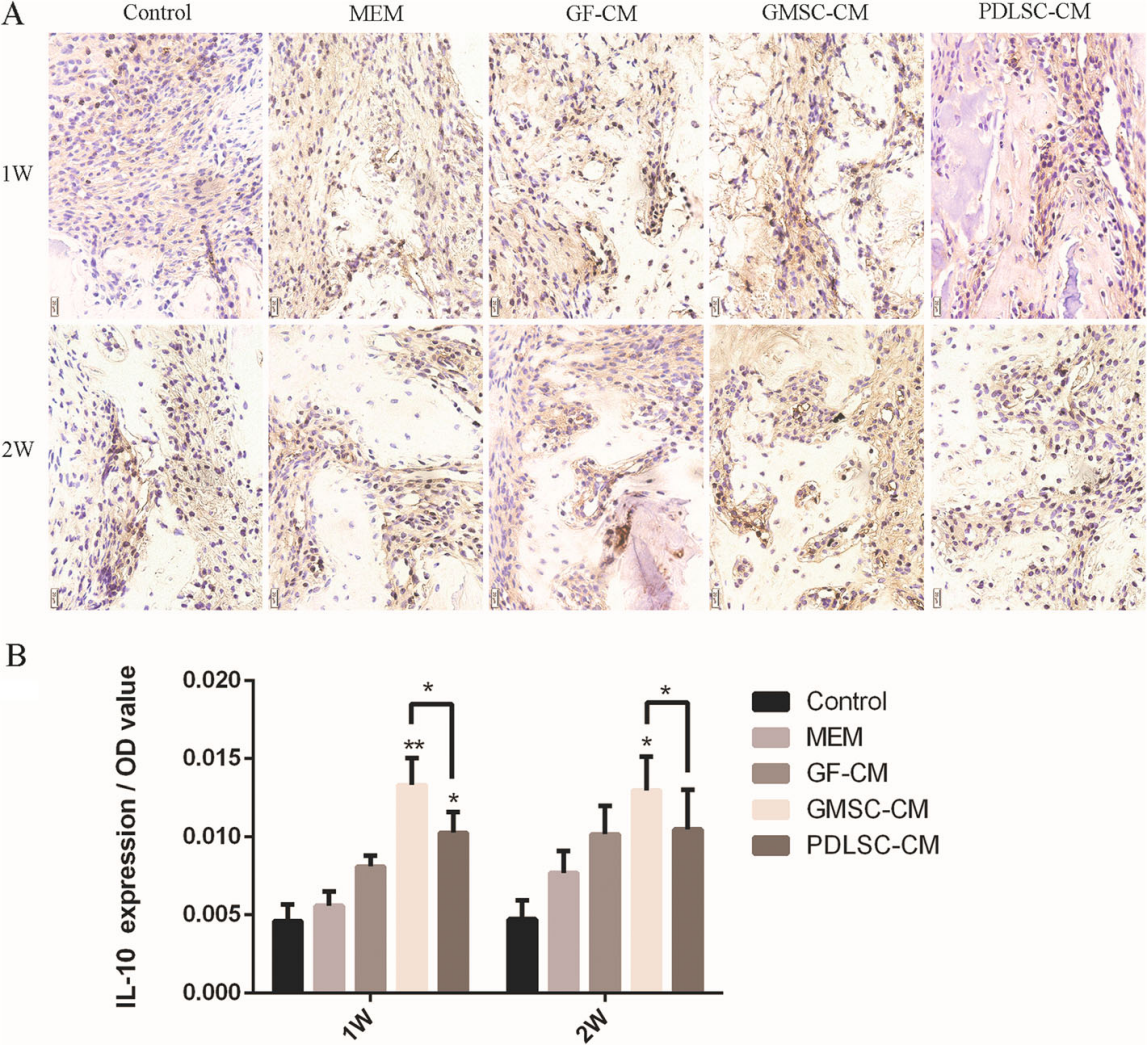
Effect of conditioned media on the osteogenesis-related marker IL-10. **A** Representative images of immunohistochemistry of IL-10 (400×) at 1 and 2 weeks. **B** Relative quantification of IL-1β immunohistochemical staining. *P < 0.05, **P < 0.01 vs. control group

PDLSC-CM has been documented to possess anti-inflammatory properties, and some studies have shown that GMSCs have superior anti-inflammatory and immunomodulatory functions [28, 29]. Wound healing and regeneration are greatly influenced by inflammation. Therefore, immunohistochemical staining for TNF-α, IL-1β, and IL-10 was used to evaluate inflammatory modulation by GMSC-CM and PDLSC-CM at the surgical periodontal site. TNF-α (Fig. 7A) and IL-1β (Fig. 8A) presented almost the same staining results, and the positively stained cells were distributed in the defect area and along the inner side of the collagen membrane. More positively stained cells were found at 1 week than at 2 weeks within each group. At both 1 and 2 weeks, the expression levels of TNF-α (Fig. 7B) and IL-1β (Fig. 8B) were significantly lower in the GMSC-CM and PDLSC-CM groups than in the other three groups, and there was no significant difference between these two groups. On the other hand, within each group, fewer IL-10-positive cells were found at 1 week than at 2 weeks (Fig. 9B). At 1 week, the GMSC-CM and PDLSC-CM groups exhibited significantly more IL-10-positive cells than the other three groups (Fig. 9B). Moreover, the GMSC-CM group showed higher expression of IL-10 than the PDLSC-CM group. At 2 weeks, the GMSC-CM group exhibited significantly higher expression of IL-10 than the other four groups. There was no significant difference between the PDLSC-CM group and the control, α-MEM, and GF-CM groups (Fig. 9B). These results suggest that both PDLSC-CM and GMSC-CM transplantation reduce inflammation in healing tissues and that GMSC-CM may have superior ability than PDLSC-CM when anti-inflammatory potential is considered.

## Discussion

The ultimate goal of periodontal treatment is to regenerate the destroyed tooth-supporting tissues to their original form, architecture, and function. The objective of the current study was to compare the regenerative potential of GMSC-CM and PDLSC-CM in a periodontal bone defect model. The results demonstrate that CMs derived from both GMSCs and PDLSCs can enhance periodontal tissue regeneration in periodontal defects created in mandibular first molars of Wistar rats. Furthermore, the effects of GMSC-CM on periodontal tissue regeneration were comparable to those of PDLSC-CM. Our results are in agreement with those of Nagata et al. [17], who showed that transplantation with a high concentration of PDLSC-CM enhanced periodontal regeneration in rats.

Previous studies have indicated that MSC-CM, which contains multiple paracrine growth factors and cytokines secreted into the culture medium by stem cells, can be used to treat various degenerative conditions, including periodontal conditions [15, 38]. The effects of MSC-CM include angiogenesis, immunomodulation, anti-apoptosis activity, cellular growth support, and chemoattraction [39–41]. Nagata et al. [17] were not the only group to apply MSC-CM to periodontal defects. Inukai et al. [38] and Kawai et al. [18] also reported that BMSC-CM promoted periodontal defect regeneration. In the above three studies, the authors used MSCs derived from different tissues. Although they all achieved periodontal tissue regeneration to some extent, the methods used to acquire MSCs were dependent on the donor resources available to the authors, and thus, we sought to identify more easily available stem cells to satisfy future applications. GMSC transplantation contributes to bone tissue regeneration, indicating that GMSCs are a promising stem cell resource for bone regeneration [31, 32]. GMSCs are a promising source of stem cells because of their regenerative and immunomodulatory properties, capacity for scarless wound healing, easy isolation, and expansion ability [42]. However, as the CMs may differ according to the cell source [15], the function of GMSC-CM in periodontal regeneration needs to be confirmed. Our results shed light on the potential widespread applications of GMSC-CM in periodontal wound healing.

To explore the mechanism of periodontal tissue regeneration enhancement by GMSC-CM and PDLSC-CM, we further studied osteogenesis in periodontal defects. The GMSC-CM group, similar to the PDLSC-CM group, showed higher expression of osteogenesis-related markers, which demonstrated that CMs from MSCs may enhance periodontal tissue regeneration by promoting the osteogenic differentiation of bone progenitor cells in the wound region. The osteogenic differentiation process includes three stages of matrix formation: proliferation, maturation, and mineralization. Runx2 protein is the first transcription factor required for the determination of osteoblast lineage; it is first detected in preosteogenic cells, upregulated in immature osteoblasts, and downregulated in mature osteoblasts [43]. BSP is mainly a bone matrix component derived from osteoblasts and is expressed in the late stage of differentiation [44]. Immunohistochemical analysis indicated that GMSC-CM significantly increased the levels of BSPII and Runx2 in periodontal defects and achieved the same effect as PDLSC-CM. We demonstrated that GMSC-CM promoted alveolar bone formation in the defect.

PDLSC-CM has been documented to possess anti-inflammatory properties, and some studies have shown that GMSCs have superior anti-inflammatory and immunomodulatory functions [28, 29]. Wound healing and regeneration are greatly influenced by inflammation. Periodontitis is clinically defined as inflammation associated with microorganisms and mediated by the host, which results in the loss of periodontal attachment. During periodontal bone resorption, inflammatory cytokines result in lymphocytes and fibroblasts producing receptor activator of nuclear factor-kappa B (RANK) and activation of monocytes, which differentiate into macrophages and preosteoclasts [45]. Moreover, inflammatory cytokines combined with the RANK ligand induce preosteoclasts to differentiate into osteoclasts [46]. Much periodontal tissue destruction is ascribed to TNF-α and IL-1 activities [47, 48]. IL-10, as an anti-inflammatory cytokine, inhibits the functions of Th1 cells; reduces the secretion of proinflammatory cytokines, such as TNF-α, IL-1, IL-2, IL-6, and IL-8; and promotes the production of protective antibodies [49]. IL-10 regulates proinflammatory cytokines and plays an important role in suppressing inflammatory and immune responses [50, 51]. In the current experiment, our immunohistochemical analysis indicated that GMSC-CM and PDLSC-CM significantly decreased the levels of TNF-α and IL-1β and increased the level of IL-10 in periodontal tissue. Moreover, GMSC-CM significantly decreased the levels of TNF-α and IL-1β, achieving the same effect as PDLSC-CM, but more significantly increased the level of IL-10 than PDLSC-CM. These results demonstrate a correlation among the suppression of TNF-α and IL-1β, the promotion of IL-10, and periodontal regeneration. These findings support the conclusion that GMSC-CM enhances periodontal tissue regeneration by anti-inflammatory and immunoregulatory effects via TNF-α and IL-1β inhibition and IL-10 promotion.

To our knowledge, this study is the first to show that GMSC-CM enhances periodontal regeneration in vivo. Furthermore, the amount of regeneration achieved with GMSC-CM was similar to that achieved with PDLSC-CM. Compared with PDLSC-CM, GMSC-CM has several advantages, including its anti-inflammatory and immunoregulatory capacities, abundant cell sources, and higher rate of cell proliferation. Compared with MSC transplantation, CM transplantation is advantageous for its lack of tumorigenicity and ease of storage and clinical application.

Several studies have reported the use of various MSC-CMs to treat human skin wounds (adipose-derived stem cell-CM) [52], foot ulcers (amniotic MSC-CM) [53], and alopecia (adipose-derived stem cell-CM) [54] without any complications. However, further safety studies of CM transplantation are necessary.

## Conclusions

Our results showed that GMSC-CM transplantation significantly promoted periodontal defect regeneration in rats and achieved the same effect as PDLSC-CM. The mechanism by which periodontal regeneration is promoted may be related to the regulation of inflammatory factors by MSC-CM and the facilitation of osteogenic differentiation of bone progenitor cells in the wound region. Thus, transplantation of GMSC-CM or PDLSC-CM is a promising approach to inducing periodontal regeneration.

## Supplementary information

**Additional file 1.** Diagram of the buccal roots and the alveolar bone between the roots of rat first molar. DR; distal root of first mandibular molar; MR: mesial root of first mandibular molar; CR; central root of first mandibular molar; red square: coronal alveolar bone between the three roots; black square: the middle part of alveolar bone between the roots.

**Additional file 2.** Newly formed tissue in GMSC-CM and PDLSC-CM groups at 1 week (200×, HE staining). High (200×) magnification of Fig. 2A. NB: new alveolar bone; D: dentin.

**Additional file 3.** Newly formed tissue was observed by Masson staining in GMSC-CM at 1 week. B1:Low (40×) magnification. B2:High (200×) magnification of the tissue in the upper square, the newly formed calcified tissue was stained blue and the it seemed a little looser than the old alveolar bone; B3: High (200×) magnification of the tissue in the lower square. The old alveolar bone was stained red. NB: new alveolar bone; D: dentin; OB:old alveolar bone.

**Additional file 4.** Newly formed tissue was observed by Masson staining in PDLSC-CM at 1 week. B1:Low (40×) magnification. B2:High (200×) magnification of the tissue in the upper square, the newly formed calcified tissue was stained blue and the it seemed much looser than the old alveolar bone; B3: High (200×) magnification of the tissue in the lower square. The old alveolar bone was stained red. NB: new alveolar bone; D: dentin; OB:old alveolar bone.

## Data Availability

The datasets used and/or analyzed during the current study are included in this published article or available from the corresponding author upon reasonable request.

## Abbreviations

BSP: Bone sialoprotein
EDTA: Ethylene diamine tetraacetic acid
FBS: Fetal bovine serum
GMSC-CM: Gingival mesenchymal stem cell conditioned medium
HE: Hematoxylin-eosin
IL: Interleukin
MSCs: Mesenchymal stem cells
PBS: Phosphate-buffered saline
PDLSC-CM: Periodontal ligament stem cell conditioned medium
RANK: Receptor activator of nuclear factor-kappa
Runx2: Runt-related transcription factor 2
TNF: Tumor necrosis factor
α-MEM: α-Minimal essential medium
