# Supplementary figures and images for: Enhancement of periodontal tissue regeneration by conditioned media from gingiva-derived or periodontal ligament-derived mesenchymal stem cells: a comparative study in rats
Source: Stem Cell Res Ther. 2020 Feb 3;11:42. doi: 10.1186/s13287-019-1546-9 (PMC6998241; doi:10.1186/s13287-019-1546-9)

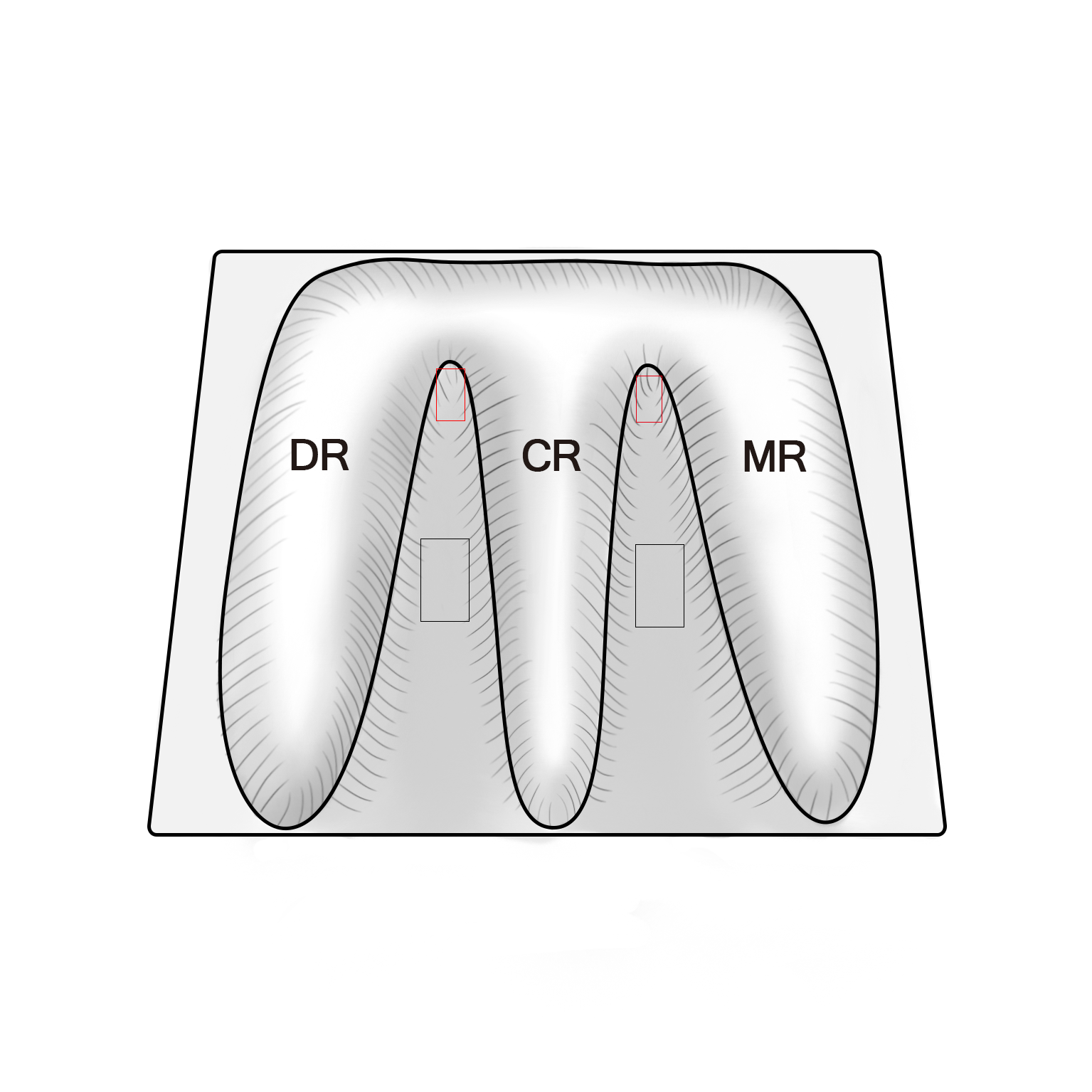

Supplement: Supplementary file 1 — Additional file 1. Diagram of the buccal roots and the alveolar bone between the roots of rat first molar. DR; distal root of first mandibular molar; MR: mesial root of first mandibular molar; CR; central root of first mandibular molar; red square: coronal alveolar bone between the three roots; black square: the middle part of alveolar bone between the roots. [file 13287_2019_1546_MOESM1_ESM.tif]

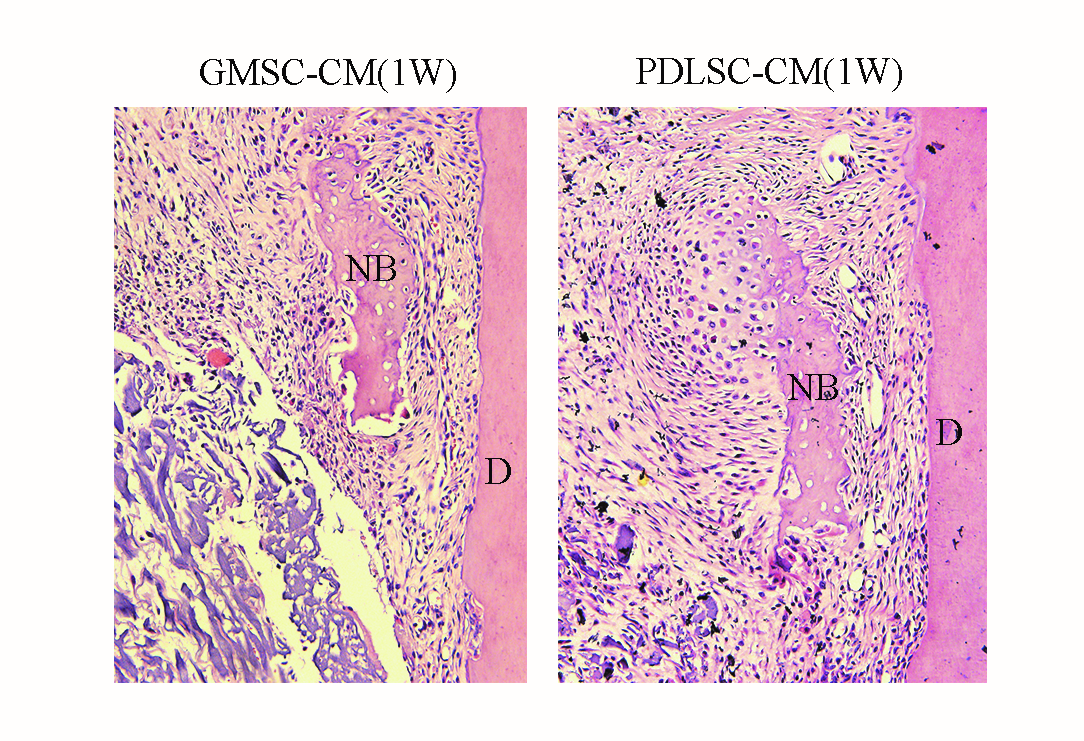

Supplement: Supplementary file 2 — Additional file 2. Newly formed tissue in GMSC-CM and PDLSC-CM groups at 1 week (200×, HE staining). High (200×) magnification of Fig. 2A. NB: new alveolar bone; D: dentin. [file 13287_2019_1546_MOESM2_ESM.tif]

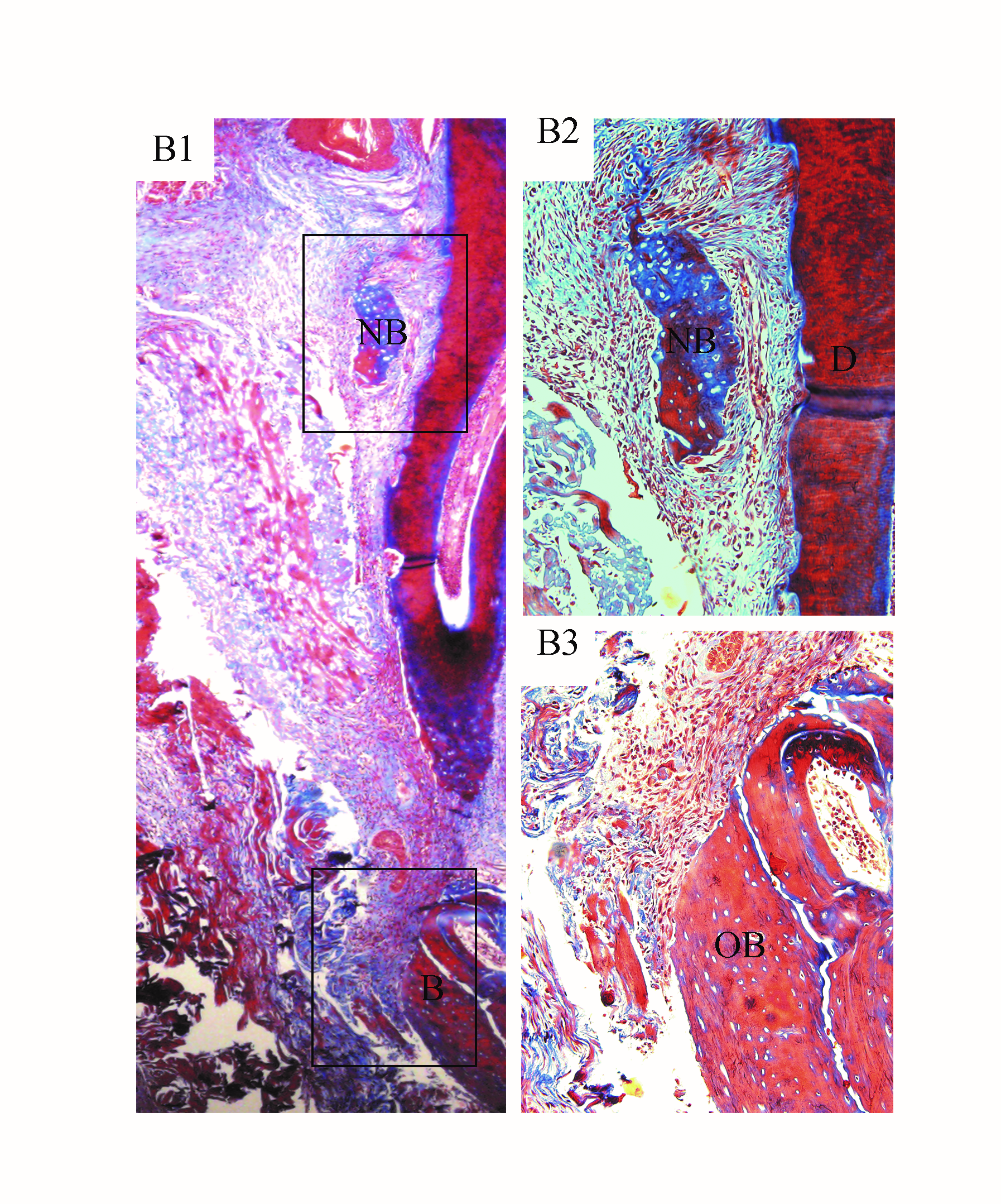

Supplement: Supplementary file 3 — Additional file 3. Newly formed tissue was observed by Masson staining in GMSC-CM at 1 week. B1:Low (40×) magnification. B2:High (200×) magnification of the tissue in the upper square, the newly formed calcified tissue was stained blue and the it seemed a little looser than the old alveolar bone; B3: High (200×) magnification of the tissue in the lower square. The old alveolar bone was stained red. NB: new alveolar bone; D: dentin; OB:old alveolar bone. [file 13287_2019_1546_MOESM3_ESM.tif]

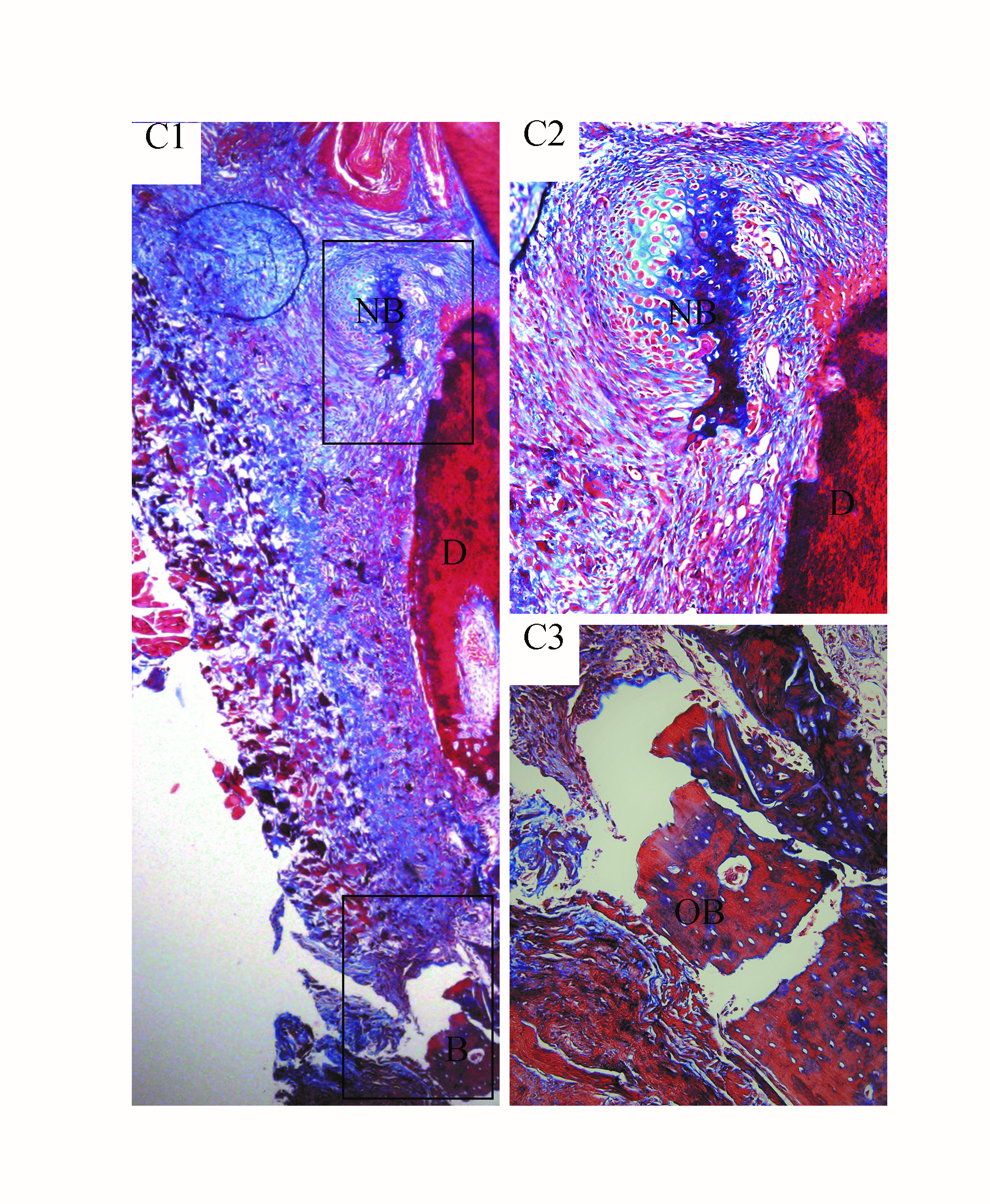

Supplement: Supplementary file 4 — Additional file 4. Newly formed tissue was observed by Masson staining in PDLSC-CM at 1 week. B1:Low (40×) magnification. B2:High (200×) magnification of the tissue in the upper square, the newly formed calcified tissue was stained blue and the it seemed much looser than the old alveolar bone; B3: High (200×) magnification of the tissue in the lower square. The old alveolar bone was stained red. NB: new alveolar bone; D: dentin; OB:old alveolar bone. [file 13287_2019_1546_MOESM4_ESM.tif]
